# Implications of a temperature increase for host plant range: predictions for a butterfly

**DOI:** 10.1002/ece3.696

**Published:** 2013-07-31

**Authors:** Hélène Audusseau, Sören Nylin, Niklas Janz

**Affiliations:** Department of Zoology, Stockholm UniversitySvante Arrhenius väg 18 B, 106 91, Stockholm, Sweden

**Keywords:** host plant range, insect–plant interactions, seasonality, specialization, voltinism shift

## Abstract

Although changes in phenology and species associations are relatively well-documented responses to global warming, the potential interactions between these phenomena are less well understood. In this study, we investigate the interactions between temperature, phenology (in terms of seasonal timing of larval growth) and host plant use in the polyphagous butterfly *Polygonia c-album*. We found that the hierarchy of larval performance on three natural host plants was not modified by a temperature increase as such. However, larval performance on each host plant and temperature treatment was affected by rearing season. Even though larvae performed better at the higher temperature regardless of the time of the rearing, relative differences between host plants changed with the season. For larvae reared late in the season, performance was always better on the herbaceous plant than on the woody plants. In this species, it is likely that a prolonged warming will lead to a shift from univoltinism to bivoltinism. The demonstrated interaction between host plant suitability and season means that such a shift is likely to lead to a shift in selective regime, favoring specialization on the herbaceous host. Based on our result, we suggest that host range evolution in response to temperature increase would in this species be highly contingent on whether the population undergoes a predicted shift from one to two generations. We discuss the effect of global warming on species associations and the outcome of asynchrony in rates of phenological change.

## Introduction

As a result of global changes in climate, species in many parts of the world are facing an increase in temperature (Hughes 2000; Parmesan and Yohe 2003; IPCC 2007). Numerous cases of phenological changes, modification in the interactions between trophic levels, range shifts, and extinction events have been documented in response to climatic changes (Parmesan 2006). Many studies have stressed the correlation between temperature and phenology (Roy and Sparks 2000; Stefanescu et al. 2003). However, the impact of temperature is more complex than a direct relationship with a species' phenology. Temperature affects species directly by modifying behavior, morphology, physiology, and overall fitness, and indirectly by modifying species interactions, habitat, and resources (Hughes 2000; Walther et al. 2002; Parmesan 2006; Preston et al. 2008; Sheldon et al. 2011).

Among the indirect effects of temperature, its impact on food resources is of major importance because of its rapid impact on the population dynamics and survival. Such effects have been documented through monitoring and field observations. Data from monitoring studies have, for example, demonstrated a mismatch between reproductive time of the European great tits and food availability (caterpillars) due to climate changes (Visser et al. 2004). With earlier onset of spring the caterpillars mature before the chicks hatch. This pattern is far from being unusual (Thackeray et al. 2010). Moreover, the response to the indirect effect of temperature varies between species. In a comparative study of a large number of insect species, Altermatt (2010b) showed that varying degrees of phenological flexibility was closely related to traits related to the life cycle, and to plant–herbivore interactions. Field observations have also demonstrated the relevance of a phenological match with food availability, especially in terms of survival (Wiklund and Friberg 2009).

Nevertheless, to our knowledge, few experimental manipulations have been done on the effect of the interaction between temperature and host plant on larval performance in a context of climatic change (Braschler and Hill 2007). Stamp and Bowers (1990) reported the effect of the combined role of host plant quality and temperature on growth rate in a context of predation risk. Kingsolver et al. (2006) experimentally showed the combined role of temperature and diet on the performance of the small cabbage white butterfly. However, this study compared natural diet to artificial diet with the objective to estimate potential biases in laboratory experiments, which often use artificial diet to investigate effects of pest insects on agriculture.

Additionally, the outcome of climatic effects on resource use can potentially be complicated through interactions with other aspects of the species' life cycle. For example, climate change can also lead to changes in the number of reproductive cycles. Many insects species may undergo a voltinism shift if the temporal window for reproduction becomes wider (Braune et al. 2008; Jönsson et al. 2009; Altermatt 2010a,b). Such an increase in the number of reproductive cycles can be particularly important because it allows faster potential population growth, making, for example, outbreaks of pest species more likely (Steinbauer et al. 2004). Individual larvae growing in different parts of the season may experience quite different environmental conditions, including food plant suitability and availability (e.g., Rausher 1980; Alonso and Herrera 2000; Rodrigues and Moreira 2004). As a consequence, voltinism shifts should be associated with shifts in patterns of host use (Nylin 1988; Scriber and Lederhouse 1992; Nylin et al. 2009; Altermatt 2010b). Indeed, some host plants may be better than others at supporting the production of an additional generation (Hunter and McNeil 1997; Wedell et al. 1997).

In order to be able to better predict the impacts of climate change, we need to understand not only how individual species react to temperature changes but also how their interactions with other species will change and how the interactions are affected by potential life cycle shifts. The polyphagous butterfly *Polygonia c-album* is known to vary in host use with voltinism and latitude (e.g., Nylin 1988; Asher et al. 2001; Nylin et al. 2009). Southern bivoltine populations tend to be more specialized on plants in the Urticaceae and related families – mainly the herb *Urtica dioica* – whereas northern univoltine populations also include a range of trees and bushes – such as *Ulmus*, *Salix*, *Betula*, and *Ribes* – in the repertoire. These differences in host use may partly be the result of phenotypic plasticity, but there is also a genetic component to the variation between populations (Janz 1998; Nygren et al. 2006).

As far as we know, *Polygonia c-album* is still strictly univoltine in Sweden (Species Gateway 2012; free reporting system for species observations in Sweden), but a partial second generation occurs further south (Tolman and Lewington 1997) and is expected to appear if bivoltinism is a plastic response to temperature and mean temperatures continue to increase. Hence, Swedish populations are soon likely to face not only increased temperatures during the time of larval growth but also an imminent shift from one to two generations. The Swedish population of this butterfly is therefore a suitable system for investigating the consequences of shifts in temperature on host plant utilization and how this interacts with a shift in voltinism, through plasticity and evolution. Mor-eover, we may have a real-world evolutionary response in a decade or two, against which our predictions can be compared.

Here, we present a study on butterflies from a Swedish population of the polyphagous butterfly, *Polygonia c-album*. Larval development is a central part of the butterfly life cycle because individuals are directly affected by the quality of its host only during that time. Thus, we investigated larval responses to temperature and host plant at two periods that would match with the feeding time of a hypothetical bivoltine population. A temperature increase is expected to loosen constraints on the host plant range and to affect the selective regime. We hypothesize that an increase in temperature will initially favor a wider host plant range, by making more plants suitable for larval growth. Indeed, while herbaceous plants produce new leaves throughout the whole season, the flushing of woody plants is simultaneous in early spring, followed by a seasonal decrease in quality as food for larvae (Feeny 1970; Cizek et al. 2006). Hence, early feeding larvae will be able to complete their development before the leaf quality of trees and bushes deteriorate too much. This pattern is likely to become more complex if a potential shift in voltinism is considered. If the temporal window for reproduction becomes wider, at least a part of the population may have an extra generation. This is the situation in England where 30–40% of the population was estimated to be bivoltine already many decades ago (Frohawk 1924), a percentage that most likely has increased since that time (Hodgson et al. 2011). In that case, the food resource available for the larvae of the second generation will be different and the leaves of herbaceous plants, *Urtica dioica*, are likely to be preferred over the leaves of woody plants.

## Materials and Methods

### Study organism

*Polygonia c-album* is a polyphagous species known to feed on seven plants families: Urticaceae, Ulmaceae, Cannabaceae, Salicaceae, Betulaceae, Corylaceae, and Saxifragaceae. However, the full range of species is not used in all populations, some are more specialized, such as the UK and Spanish, and some use the entire repertoire, such as the Swedish (Nylin et al. 2009). These differences between populations have a genetic basis (Janz 1998; Nygren et al. 2006). In our experiment, we used high-, middle-, and low-preferred plants in the rank order; *Urtica dioica*, *Salix caprea*, and *Betula pubescens,* respectively. Fresh leaves of the herbaceous species *U. dioica* are available all along the season while the leaf quality of the woody plants, *Betula pubescens* and *Salix caprea,* are expected to decrease through the season (Feeny 1970; Alonso and Herrera 2000; Salminen et al. 2001; Scheirs et al. 2002). Throughout its geographic range, different populations of *P. c-album* can be uni- to multivoltine. Differences in adult color morphs between diapausing and directly reproducing individuals indicate differences in developmental pathway. Nonetheless, the study population is univoltine.

### Experimental protocol

Mated adult females were wild caught from four different localities around the Stockholm area at the beginning of the reproductive cycle (end of April). Adult females were placed in individual cages (about 0.5*0.5*0.5 m) under 7L:17D photoperiod and a temperature of 23.5 ± 1.5°C, and humidity was maintained in the cages with wet paper. Adults were fed with sugar water and host plants were placed in each cage to allow oviposition. Eggs were collected daily and saved before hatching in small boxes, under natural light and room temperature.

At total of 399 eggs from five identified females were used. We considered this initial sample size to be sufficient based on the results from Nylin et al. (2005) showing that much of the regional genetic variation linked to host plant preferences can be found in any local population.

Upon hatching, larvae were randomly transferred individually in a 2 × 3 factorial design, consisting of three food plants (*U. dioica*, *B. pubescens*, or *S. caprea*) and two temperature regimes (constant temperature of 15.1 ± 0.4 or 23.2 ± 0.4°C). The temperatures chosen correspond to the range of temperature the larvae can experience in Sweden during the reproductive cycle. Larvae were reared individually in small plastic cups and fed ad libitum with fresh leaves collected from the field. Leaf quality was maintained using a moistened sponge in the bottom of the cups, and leaves were replaced daily or every second day. Larvae were kept at 12L:12D photoperiod.

Mass and date of newly molted fifth instar larvae and pupae were recorded as well as emergence date. Sex was determined at pupal stage. Upon adult emergence of individuals of the first generation, individuals were sorted according to family and sex (to avoid inbreeding), and placed in separate cages to mate. Due to the short day length used during the larval rearing, dark morphs – or diapausing adults – were obtained. Dark morph adults are normally univoltine, but can be manipulated to reproduce without diapause by exposing them to a relatively high temperature (26.2 ± 1.5°C) and long day length (22L:2D).

In total, 1210 eggs from 10 females were used in the second experiment. Mated females were placed in the same conditions as the wild-caught females for egg laying. Egg collection, storage until hatching, larval rearing, and data collection were done as described for the first brood.

### Statistical analysis

All statistical analyses were performed in the software R, version 2.13.1 (R Development Core Team 2011). We used the functions *lmer* and *glmer* in the R package lme4 (Bates et al. 2011) to investigate the responses of different measures of larval performance to temperature and host plant. The measurements of mass and development time (fifth instar larvae, pupae), pupal development time, and the logarithm of the growth rate (eq. 1) were used as performance measures. Growth rate is a measure of mass gain. The use of growth rate minimizes undesirable variations due to the protocol (larval stage and mass were recorded only once a day) and it is known to be a relevant measurement of fitness, well correlated with oviposition preference at the population level (Janz et al. 1994). A total of 51 neonate individuals used in the experiment were weighed to estimate mean neonate mass, which was 0.231 mg.


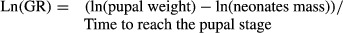
(1)

For each reproductive event – or brood – models were calibrated using the effects of temperature (*T*), host plant (P), sex, and the two-way interactions between these variables as fixed effects (written as fixed ∼ *T* + P + sex + *T*: P + *T*: sex + P: sex). Models included the effect of family and its interaction with temperature and host plant as random factors (random∼1 + H + *T* | family).

Then, we investigate the effect of host plant seasonality on larval performance measures using the complete dataset (data from both broods were compiled). The effects of temperature, host plant, brood, sex, and the two-way interactions on larval performance were included as fixed effects. Similar to the analyses within brood, the effect of family and its interaction with temperature and host plant was included in the models as random factors.

Model fitting followed the procedure proposed by Bolker et al. (2009). The selection for the random effects was performed by doing a step-by-step backward procedure with fixed effects kept constant. Model selection was based on differences in AIC calculated using the ML (Nakagawa and Schielzeth 2012). The most parsimonious model still included the family effect because it was part of the experimental design. The selection for the interactions among fixed effects was performed by doing a step-by-step backward procedure as for the random effects. Likewise, model selection was based on differences in AIC calculated using the ML. Main effects of fixed factors were never dropped since they were part of the experimental design.

The performance measures mass at fifth instar and pupal mass were log transformed to get closer to normality. The distributions of model residuals for the other performance measures did not always match normality but considering the large size of our sample (at least 258 individuals), the good fit of the models selected, and after checking for overdispersion we consider the potential bias nonsignificant.

Additionally, we calculated a measure of explained variation due to the fixed effects' *R*² following the method proposed by Nakagawa and Schielzeth (2012).

Finally, we tested the fixed effects of temperature, host plant, brood, and the two-way interactions on larval survival rates. Family was included as a random factor. We performed a generalized linear mixed model using a quasibinomial distribution (logit-link function) to account for the overdispersion. Model selection was similar to the one for the other performance measures.

## Results

The measure of larval growth rate, both broods considered, varied with temperature, host plant, brood (which measures the seasonality, or a temporal shift in reproduction), and all two-way interactions (Table 1). The model selected fitted the data closely (*R*^2^ = 93.20, Table 2). The impact of temperature, host plant, and the interaction between temperature and host plant remained for the models selected when considering each brood separately (Tables 2 and 3).

**Table 1:** Analysis of deviance table (type II Wald Chi-square tests) showing the effect of temperature (*T*), host plant (P), seasonality (Brood), and the two-way interactions between those variables on larval growth rate (*N* = 829)

Growth rate	df	Chisq	*P*
*T*	1	2575.99	<0.0001
P	2	2306.97	<0.0001
Brood	1	288.94	<0.0001
Sex	1	0.0283	0.87
P × *T*	2	686.94	<0.0001
P × Brood	2	11.68	0.0029
*T* × Brood	1	183.32	<0.0001

Brood, temperature, host plant, and the two-way interactions in the table were considered as fixed effects and family as a random effect of the mixed model.

**Table 2:** Factors affecting the different performance measures. Between broods, we tested the effect of temperature (*T*), host plant (P), seasonality (Brood), sex, and the two−way interactions on larval performance measures (mass and development time fifth instar larvae and pupae, pupal time and growth rate). Within brood, we tested the effect of temperature, host plant, sex, and the two−way interactions. Those analyses allowed us to investigate, respectively, inter− (seasonal) and intrabrood variations. The *N* values indicate the sample sizes

Performance measure	*N*	Data	Linear model selected	AIC	*R*^2^
Log mass 5th instar	812	Brood 1 + 2	Ln mass5 ∼ ***T*** + **P** + **Brood** + Sex + **Brood** × ***T***	−196.4	13.94
	284	Brood 1	Ln mass5 ∼ ***T*** + **P** +Sex + **Sex** × **P**	18.76	12.86
	528	Brood 2	Ln mass5 ∼ ***T*** + **P** + Sex	−208.4	6.56
Log mass pupae	836	Brood 1 + 2	Mass p ∼ ***T*** + **P** + **Brood** + **Sex** + **P** × ***T*** + **Brood** × **P** + **Brood** × ***T***	−1309	18.46
	303	Brood 1	Mass p ∼ ***T*** + **P** + **Sex** + ***T*** × **Sex**	−487.2	32.17
	533	Brood 2	Mass p ∼ ***T*** + P + **Sex** + **P** × ***T***	−821	8.06
Development Time to reach the 5th instar	829	Brood 1 + 2	DT 5 ∼ ***T*** + **P** + **Brood** + Sex + **P** × ***T*** + **Brood** × **P**	4149	91.02
	301	Brood 1	DT 5 ∼ ***T*** + **P** + Sex + **P** × ***T***	1441	93.53
	528	Brood 2	DT 5 ∼ ***T*** + **P** + Sex + **P** × ***T***	2703	92.43
Development Time to reach the pupal stage	829	Brood 1 + 2	DT p ∼ ***T*** + **P** + **Brood** + Sex + **P** × ***T***	2721	89.52
	301	Brood 1	DT p ∼ ***T*** + **P** + Sex + **P** × ***T***	984.6	89.89
	528	Brood 2	DT p ∼ ***T*** + **P** + Sex + **P** × ***T***	1742	89.15
Pupal time	654	Brood 1 + 2	DT a ∼ ***T*** + **P** + **Brood** + Sex + **P** × ***T*** + **Brood** × ***T*** + **Sex : H**	2444	94.4
	258	Brood 1	DT a ∼ ***T*** + **P** + Sex+ **P** × ***T*** +***T*** × **Sex**	1058	91.8
	396	Brood 2	DT a ∼ ***T*** + Family + Sex	1338	96.45
Growth Rate	829	Brood 1 + 2	GR ∼ ***T*** + **P** + **Brood** + Sex + **P** × ***T*** + **Brood** × ***T*** + **Brood** × **P**	−3597	93.20
	301	Brood 1	GR ∼ ***T*** + **P** + Sex + **P** × ***T***	−1291	94.17
	528	Brood 2	GR ∼ ***T*** + **P** + Sex + **P** × ***T***	−2330	94.50

Temperature, host plant, brood, and sex were considered as fixed effects and family as a random effect of the mixed model (see text for more details). For size models, Akaike Information Criterion (AIC) values were calculated using maximum likelihood (ML) and *R*² from restricted maximum likelihood (REML) estimations. The *R*² gives information on the variance explained by the fixed effect. In bold are the significant variables from the anova tables of the selected models.

**Table 3:** Values of the different performance measures in each treatment (mean ± SD). Mass fifth instar larvae (Mass 5), development time to reach the fifth instar (DT5), mass pupae, and development time to reach the pupal time (DTp)

Treatment/Generation		G 1				G 2			
		
*T*	Host plant	Mass 5	DT5	Mass pupae	DTp	Mass 5	DT5	Mass pupae	DTp
15°C	*Betula pubescens*	103.3 ± 24.0	39.5 ± 5.2	280.2 ± 30.5	14.7 ± 1.4	96.5 ± 14.5	48.4 ± 6.1	277.9 ± 27.9	16.7 ± 2.1
	*Salix caprea*	97.1 ± 22.1	27.8 ± 2.8	299.8 ± 33.6	11.1 ± 1.7	87.1 ± 13.7	31.3 ± 3.7	287.3 ± 32.4	12.6 ± 1.2
	*Urtica dioica*	112.3 ± 26.9	23.9 ± 1.5	317.2 ± 27.5	9.5 ± 0.8	100.8 ± 16.8	25.2 ± 2.2	299.0 ± 33.6	10.7 ± 1.3
23°C	*Betula pubescens*	112.0 ± 23.5	15.1 ± 1.9	254.5 ± 23.6	7.3 ± 1.4	99.1 ± 17.1	22.7 ± 3.0	281.2 ± 29.9	8.6 ± 1.2
	*Salix caprea*	108.4 ± 26.4	11.9 ± 1.6	282.7 ± 31.1	4.9 ± 0.9	93.8 ± 20.6	14.3 ± 1.7	282.7 ± 30.8	6.1 ± 1.0
	*Urtica dioica*	132.6 ± 35.1	10.4 ± 0.9	302.5 ± 32.4	4.1 ± 0.8	106.4 ± 26.9	11.4 ± 1.0	274.7 ± 24.4	5.1 ± 0.6

The models selected for the measures of mass at the fifth instar and the pupal stage, and the development time to the same stages, presented similarities with the model selected for the larval growth rate (Table 3). The interaction between host plant and temperature remained for the majority of the performance measures except for the measure of mass at fifth instar and mass of pupae from the first brood. The effect of temperature and host plant remained on pupal duration using the complete data set. However, we think that growth rate best illustrated larval performance differences between treatments. Growth rate, as a composite measure of development time and mass, reflects the trade-off between sacrificing size and fecundity for development time or the inverse (Janz et al. 1994).

**Figure 1:**
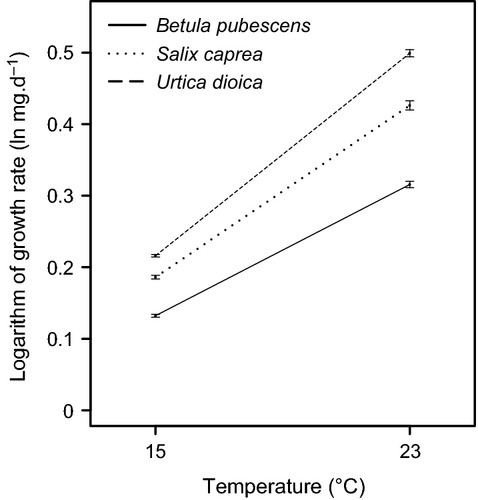
Logarithm of the growth rate of larvae from the first brood is affected by temperature and host plant (*N* = 305, mean ± SE). The variance between slopes' coefficients reflects the effect of the interaction between temperature and host plant on larval growth rate.

**Figure 2:**
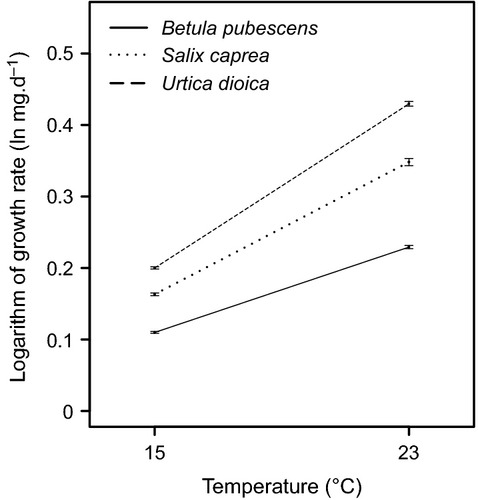
Logarithm of the growth rate of larvae from the second brood is affected by temperature and host plant (*N* = 574, mean ± SE). The variance between slopes' coefficients reflects the effect of the interaction between temperature and host plant on larval growth rate.

Figures 1 and 2 illustrate the variation in growth rate among treatments. A hierarchy in host plant suitability for larval growth was observed and this hierarchy was conserved between temperature treatments and broods. The growth rate was higher on *U. dioica*, intermediate on *S. caprea*, and lowest on *B. pubescens*. Moreover, a lower temperature seemed to be a major constraint on growth. Indeed, larval growth rates on the three food plant treatments at 15°C were closer to each other, regardless of the brood, while at 23°C the growth rate between plant treatments were more differentiated. This suggests the existence of a minimal growth rate under which the larvae cannot complete their cycle and the fact that plasticity can only be expressed when the constraint of temperature is released.

**Figure 3:**
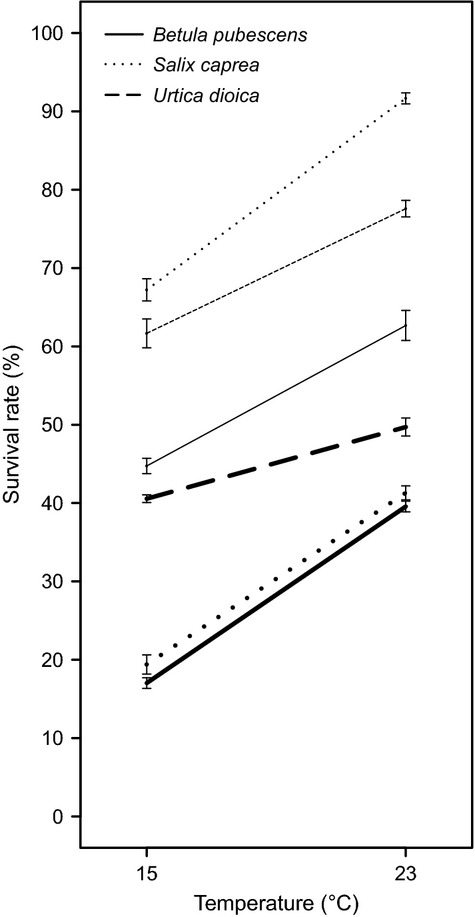
Effect of temperature, host plant, and seasonality (first brood and second brood in bold) on larval survival rate (mean ± SE). The standard errors reflect interfamily variations in larval survival. Notice the decrease in survival rate in every treatment between the first and the second brood.

Larval survival rates significantly varied with temperature, host plant, brood, and the two-way interactions between host plant and both temperature and brood (Table 4). A first observation was that a higher temperature increased survival rates independently of the host plant or the brood and that survival rates drastically dropped in all treatments for the second brood (Fig. 3).

**Table 4:** Analysis of deviance table (type II Wald Chi-square tests) showing the effects of temperature (*T*), host plant (P), and brood on larval survival (total number of larvae reared = 1562). The survival variable was built as a two-vector response variable including both the number of failures (dead larvae), and the number of successes (larvae that reached the adult stage) for each combination of temperature, host plant, brood, and family (*N* = 90). A generalized linear mixed model was fitted using a quasi-binomial error

Survival	df	Chisq	*P*
Brood	1	87.29	<0.0001
*T*	1	60.45	<0.0001
P	2	31.30	<0.0001
P × *T*	2	7.52	0.0232
P × Brood	2	14.65	0.0007

Brood, temperature, host plant, and the two-way interactions in the table were considered as fixed effects and family as a random effect of the mixed model.

Interestingly, the hierarchy in host plants suitability for larvae to survive within generation was similar between temperature treatments but differed between generations (Table 4, Brood × H, χ^2^(2) = 14.65, *P* = 0.0007). Individuals from the first brood had a better survival on *S. caprea*, followed by *U. dioica*, and then *B. pubescens* (Fig. 3). Individuals from the second brood feeding on *U. dioica* survived better than individuals feeding on the two woody plants (Fig. 3).

The drop of the slopes between broods, for each food plant treatment (Fig. 3), stresses the effect of phenology that affected woody plants more seriously. Moreover, survival rates of the second brood larvae feeding on *S. caprea* and *B. pubescens* were more strongly affected by low temperature than larvae feeding on *U. dioica*.

## Discussion

As expected, both temperature and host plant affected larval performance and survival. Higher temperature allowed faster larval development and growth, and higher survival. All performance measures were affected by host plant. The composite measure of growth rate was highest on *U. dioica*, and lowest on *B. pubescens*, which is consistent with previous results (Janz et al. 1994). This hierarchy did not change with temperature or with seasonal progression despite that growth rates were lower overall for the second brood. Larval survival responded somewhat differently to host plants. Early in the season, survival was highest on *S. caprea* and lowest on *B. pubescens*. We observed a change in host plant rank with seasonal progression with the herbaceous *U. dioica* allowing higher survival rates than woody plants.

Moreover, our results confirmed the role of the interaction between host plant and temperature in larval performance that has previously been seen in this species (Janz et al. 1994; Braschler and Hill 2007) and other butterfly species (Stamp and Bowers 1990; Kingsolver et al. 2006). Growth rate increase was steeper in the herbaceous plant when temperature increased than on woody plants for both broods. Our results show that this holds comparing a range of wild host plants but also that the strength of this interaction increases through the season. This can probably be explained by the fact that another constraint on larval development is added during the second reproductive event: the decrease in leaf quality that is affecting woody plants more strongly. It is not surprising that larval performance in every treatment is affected by seasonal progression, and that larvae feeding on woody plants are more affected than larvae feeding on herbaceous plants. The relative quality of the trees is dramatically reduced over the summer in terms of water content, C:N ratio, and accumulation of tannins (Feeny 1970; Alonso and Herrera 2000; Scheirs et al. 2002).

This result is particularly interesting in the light of the finding that species with herbaceous larval food plants are more likely to shift in voltinism if the temporal window for reproduction becomes wider (Cizek et al. 2006; Altermatt 2010b). This can be explained by the fact that herbaceous plants have sequential leaf regrowth providing palatable leaves throughout the growing season.

A future shift in voltinism for Swedish *P. c-album* is not unlikely if mean temperatures continue to increase. For example, a proportion of the UK population of *P. c-album* is bivoltine even though the difference in mean monthly temperature is only a few degrees compared to Sweden. In the hypothetical case of the occurrence of a second brood in Sweden, our results suggest that lower larval survival on woody plants would be observed. The advantage of growing faster when feeding on *U. dioica* would be reinforced later in the season by a higher chance to survive and reach maturity in time for hibernation, Under such a scenario, the seasonal decline in leaf quality means that following a shift in voltinism, relative host plant suitability is likely to differ between generations, leading to a rapid shift in selective regime. An added generation would accelerate potential population growth, and investing in a second generation should be highly adaptive as soon as conditions so allow. As a consequence, under these circumstances there will be strong selection to specialize on the plants that can support the highest growth rate – and that can sustain it throughout the season – in order to complete the additional generation (cf. Nylin 1988; Scriber and Lederhouse 1992; Scriber 2002; Nylin et al. 2009).

The main implication of this study is a two-step evolutionary response to climatic change in the degree of host plant specialization of *P. c-album*. The first response to warmer temperatures would be to relax the constraint of temperature on growth rate because all the plants will support growth above the minimal threshold. Moreover, a temperature increase may allow the species' reproductive cycle to be completed within a time when differences in leaf quality between herbs and trees are less pronounced. Second, if the climatic change persists and the reproductive season becomes longer, this is likely to result in a shift in voltinism. This will lead to a shift in selection regime that may initiate an evolutionary specialization on *U. dioica* because of the time constraint to achieve two reproductive cycles.

Indeed, most phytophagous insects are specialists (e.g., Schoonhoven et al. 2005). Thus, in the long-term evolution seems to favor specialization, but there are still some polyphagous exceptions that need to be explained. *P. c-album* is one such potentially polyphagous species, varying in actual degree of specialization as well as belonging to a genus of butterflies where there are also many specialists, suggesting a dynamic picture of evolutionary changes in host range (Janz et al. 2001; Weingartner et al. 2006; Dennis et al. 2011).

The scenario of response to climate warming that we propose helps in providing an explanation to these rapid movements along the specialization gradient. The process of host plant specialization can be driven by both external (abiotic conditions, plant distribution/availability/quality) and internal factors (life cycle regulation), and may be quite rapid (cf. Thompson 1998). The potential rapidity of the adaptation to climate warming suggests that initial responses are predominantly plastic, but with time evolutionary changes are likely to increase in importance. The theoretical work done by Lande (2009) gives support to such a scenario. Interestingly, the direction of the change is not straightforwardly predictable by simple extrapolation, as it depends on a complex interaction between the length of the available season, local temperature during larval growth, and potential shifts in voltinism. We suggest that these interactions have been involved in the past processes leading to the current degree of specialization of southern populations of *P. c-album* on *U. dioica*. Indeed, many bivoltine populations of *P. c-album* are feeding almost exclusively on *U. dioica* and the related herb *Humulus lupulus* (Nylin et al. 2009).

In conclusion, insects have been shown to extend their ranges northwards as a consequence of warming (Parmesan et al. 1999), sometimes leading to changes in patterns of voltinism (Altermatt 2010a). Based on our results, we suggest that warming can also affect patterns of host use, and in particular host plant range. In an applied perspective, the interaction between temperature, specialization, and voltinism may have significant and sometimes unexpected consequences as pest species colonize higher latitudes and/or increase their number of reproductive cycles (Steinbauer et al. 2004; van Asch and Visser 2007; Martin-Vertedor et al. 2010).
